# Mutational Analysis of the Ve1 Immune Receptor That Mediates *Verticillium* Resistance in Tomato

**DOI:** 10.1371/journal.pone.0099511

**Published:** 2014-06-09

**Authors:** Zhao Zhang, Yin Song, Chun-Ming Liu, Bart P. H. J. Thomma

**Affiliations:** 1 Laboratory of Phytopathology, Wageningen University, Wageningen, The Netherlands; 2 Key Laboratory of Plant Molecular Physiology, the Chinese Academy of Sciences, Beijing, China; Virginia Tech, United States of America

## Abstract

Pathogenic *Verticillium* species are economically important plant pathogens that cause vascular wilt diseases in hundreds of plant species. The *Ve1* gene of tomato confers resistance against race 1 strains of *Verticillium dahliae* and *V. albo-atrum*. *Ve1* encodes an extracellular leucine-rich repeat (eLRR) receptor-like protein (RLP) that serves as a cell surface receptor for recognition of the recently identified secreted *Verticillium* effector Ave1. To investigate recognition of Ave1 by Ve1, alanine scanning was performed on the solvent exposed β-strand/β-turn residues across the eLRR domain of Ve1. In addition, alanine scanning was also employed to functionally characterize motifs that putatively mediate protein-protein interactions and endocytosis in the transmembrane domain and the cytoplasmic tail of the Ve1 protein. Functionality of the mutant proteins was assessed by screening for the occurrence of a hypersensitive response upon co-expression with Ave1 upon *Agrobacterium tumefaciens*-mediated transient expression (agroinfiltration). In order to confirm the agroinfiltration results, constructs encoding Ve1 mutants were transformed into Arabidopsis and the transgenes were challenged with race 1 *Verticillium*. Our analyses identified several regions of the Ve1 protein that are required for functionality.

## Introduction

In order to activate immune responses that ward off invading microorganisms, plants utilize various types of receptors that recognize pathogen(-induced) ligands of various nature [1], [2]. Appropriate recognition of these ligands by the immune receptors is crucial for the activation of immune responses. These immune receptors are either extracellular cell surface receptors that detect (conserved) pathogen-associated molecular patterns (PAMPs) or damage-associated modified self-patterns, or cytoplasmic receptors that recognize highly specific pathogen effectors either directly, or indirectly through recognition of their activities [3], [4]. Both types of receptors may activate an hypersensitive response (HR), which is a rapid cell death surrounding the infection site that is thought to prevent further pathogen invasion [5].

The *Verticillium* genus comprises vascular pathogens that cause Verticillium wilt diseases in over 200 plant species worldwide [6], [7]. In tomato, immunity against *Verticillium* wilt is governed by the immune receptor Ve1 that recognizes the secreted *Verticillium* effector Ave1 [8], [9]. *Ve1* encodes a putative plasma membrane-localized extracellular leucine-rich repeat (eLRR)-containing cell surface receptor of the receptor-like protein (RLP) class [10]. Typically, the amino acid sequence of RLPs is composed of a signal peptide (SP), an eLRR domain that is shielded by N-terminal and C-terminal eLRR-caps, a single-pass transmembrane (TM) domain, and a short cytoplasmic tail that lacks obvious motifs for intracellular signaling. In some cases, an acidic domain is present between the eLRR domain and the TM domain. Furthermore, the eLRR domain can be subdivided into three domains in which a non-eLRR island or C2 domain interrupts the C1 and C3 eLRR regions [11], [12]. As RLPs lack an obvious domain for intracellular signaling, they presumably form a complex with other proteins, such as receptor-like kinases, to respond to ligand binding and initiate an immune response [11]. Indeed, it was recently demonstrated that interaction of Ve1 with the SUPPRESSOR OF BIR1 (SOBIR1) receptor-like kinase is required for Ve1-mediated immunity [13], [14].

It is conceivable that the eLRR domain of cell surface receptors acts as ligand sensor [15]. This similarly holds true for the eLRRs of Toll-like receptors (TLRs) that act in animal innate immunity [16]. The typical plant eLRR consensus motif comprises 24 amino acids, xxLxxLxxLxxLxLxxNxLt/sGxIP, where (x) represents any amino acid and (L) is sometimes substituted by other hydrophobic residues. For plants, the first eLRR protein crystal structures were resolved for a polygalacturonase-inhibiting protein (PGIP) [17], the brassinosteroid receptor brassinosteroid-insensitive 1 (BRI1) [18]–[20] and the flagellin receptor flagellin-sensitive 2 (FLS2) [21]. These studies revealed that successive eLRRs align in parallel to form a curved, slightly twisted “horseshoe-like” structure, in which parallel core β-strands (xxLxLxx) form the concave (inner) side of the protein and various helices, short β-strands and additional connecting residues form the convex (outer) side [15]. The concave side of the eLRR is thought to serve for ligand binding, where the hydrophobic (L) residues in the β-sheet (xxLxLxx) are involved in the framework that determines the overall shape of the protein, and the five variable, solvent exposed residues (x) of the β-strands determine ligand binding specificity [15]. Crystallographic analysis of PGIP demonstrated that the solvent exposed residues on the concave β-sheet surface determine the interaction with polygalacturonases [17]. Furthermore, the recently released crystal structure of BRI1 showed that the brassinosteroid hormone binds to a groove in between the concave β-sheet surface and the island domain [18]–[20]. Similarly, the conserved N-terminal epitope of bacterial flagellin (flg22) binds to the inner concave surface of the FLS2 LRR solenoid [21].

In the majority of studied eLRR receptors, ligand specificity is determined by the C1 domain [11]. We recently carried out domain swaps between Ve1 and its non-functional homolog Ve2, and demonstrated that the chimeras in which the first thirty eLRRs of Ve1 were replaced with those of Ve2 remained able to activate *Verticillium* resistance [13]. However, the C3 domain and C-terminus of Ve2 appeared not to be functional [13]. Potentially, the non-functional Ve2 receptor still interacts with the Ave1 elicitor in the C1 domain, but fails to activate immune signaling due to a non-functional C3 domain and C-terminus. Nevertheless, similar to Ve1, Ve2 still interacts with the receptor-like kinase SOBIR1 [13]. To further determine the role of eLRRs of Ve1 in ligand specificity and signal transduction, we employed a high-throughput alanine scanning mutagenesis strategy to mutate solvent exposed residues on the concave surface of each eLRR repeat of Ve1 in this study.

## Results

### Alanine scanning of the concave side of the Ve1 eLRR domain

**Figure 1:**
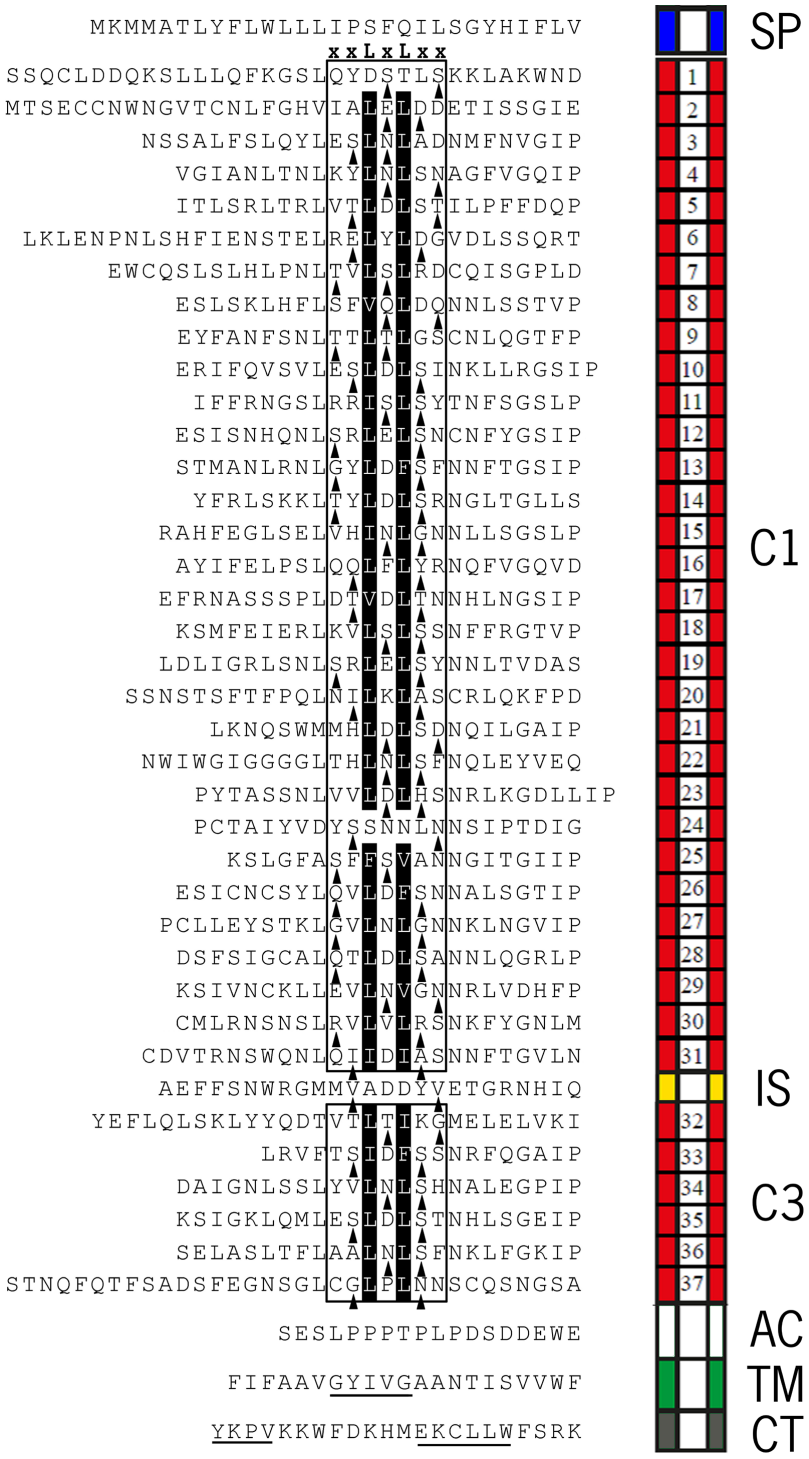
Primary structure of the Ve1 protein. Alignment of the amino acid sequence of Ve1 with a schematic representation of the protein structure. Ve1 is composed of a signal peptide (SP), eLRR region C1 (C1), island domain (IS), eLRR region C3 (C3), acidic domain (AC), transmembrane domain (TM) and cytoplasmic tail (CT). Double alanine scanning was performed on the solvent exposed β-strand residues across the Ve1eLRR domain. The putative parallel β-strands (xxLxLxx) on the concave surface are boxed, and the conserved hydrophobic residues on the concave β-sheet surface are indicated with black shading. Triangles represent solvent-exposed amino acid residues (x) subjected to alanine substitution for each of the repeats. Only one eLRR was mutated per mutant allele. The putative GxxxG motif and endocytosis signals are underlined.

Considering the large size of the Ve1 eLRR domain and avoiding the potential inefficiency of random mutagenesis, a site-directed mutagenesis strategy was performed to identify functional regions of the Ve1 eLRR domain which contains 37 imperfect eLRRs. To this end, solvent exposed residues in the β-strand of each eLRR repeat were mutated. In total, 37 mutant *Ve1* alleles were engineered, named M1–M37 respectively, in which two of the five variable solvent exposed residues in the xxLxLxx consensus of a single eLRR were mutated such that they were substituted by alanines (Figure 1). To generate mutant alleles, the *Ve1* coding sequence was cloned into pDONR207 (Invitrogen, Carlsbad, California) through a Gateway BP reaction to generate entry vector pDONR207::Ve1. Using pDONR207::Ve1 as template, and inverse PCR was performed to establish alanine substitutions by changing wild type codons in the primer sequence. The mutated *Ve1* variants were sequenced and subsequently cloned into an expression construct driven by the constitutive CaMV35S promoter.

### C1 domain eLRRs 1 to 8 and 20 to 23 are required for Ve1 functionality

**Figure 2:**
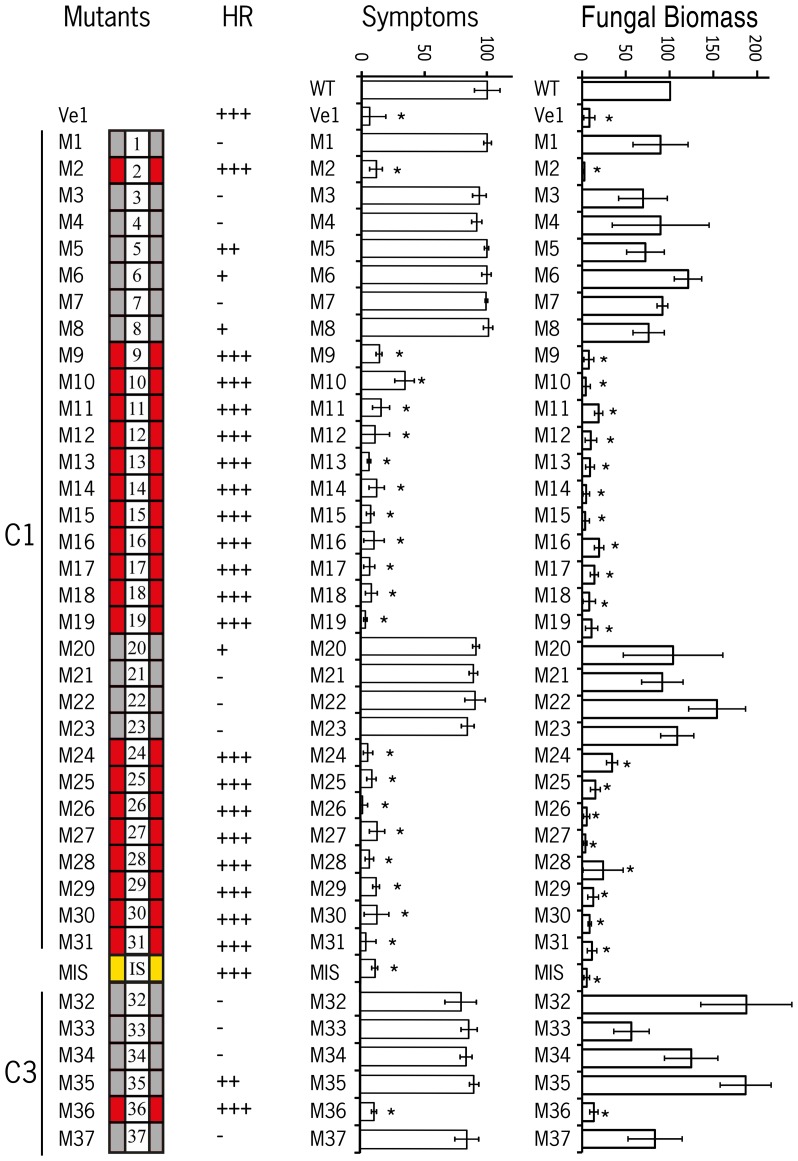
Double alanine scanning reveals eLRRs required for Ve1 functionality. A schematic representation of the Ve1 eLRR domain is shown with a summary of the functionality of the double alanine scanning mutant alleles. Grey boxes indicate mutant alleles that compromise Ve1 functionality while red boxes indicate mutants that remain fully functional. The occurrence of HR upon co-expression of Ve1 mutant alleles with Ave1 is provided, where +++ corresponds to an HR that is similar to the HR induced by wild type Ve1; ++ corresponds to an HR that is reduced when compared with the HR induced by wild-type Ve1; + corresponds to a limited HR; and - corresponds to absence of a detectable HR. Quantification of Verticillium wilt symptoms in wild type (WT) and transgenic lines is indicated. Bars represent quantification of symptoms presented as percentage of diseased rosette leaves with standard deviation with WT set to 100%. Asterisks indicate significant differences when compared with WT (P<0.001). Quantification of *Verticillium* biomass in Arabidopsis expressing Ve1 mutantconstructs is shown. Fungal biomass is determined by real-time qPCR in wild-type (WT) Arabidopsis and transgenic lines, and the fungal biomass in WT plants is set to 100%. For qPCR, *Verticillium* internal transcribed spacer (ITS) transcript levels were determined relative to Arabidopsis RuBisCo transcript levels for equilibration. Bars represent an average *Verticillium* quantification of three independent transgenic lines. Error bars represent standard deviations of qPCR results from three independent transgenic lines. Asterisks indicate significant differences when compared with WT (P<0.05). Data from a representative experiment are shown.

**Figure 3:**
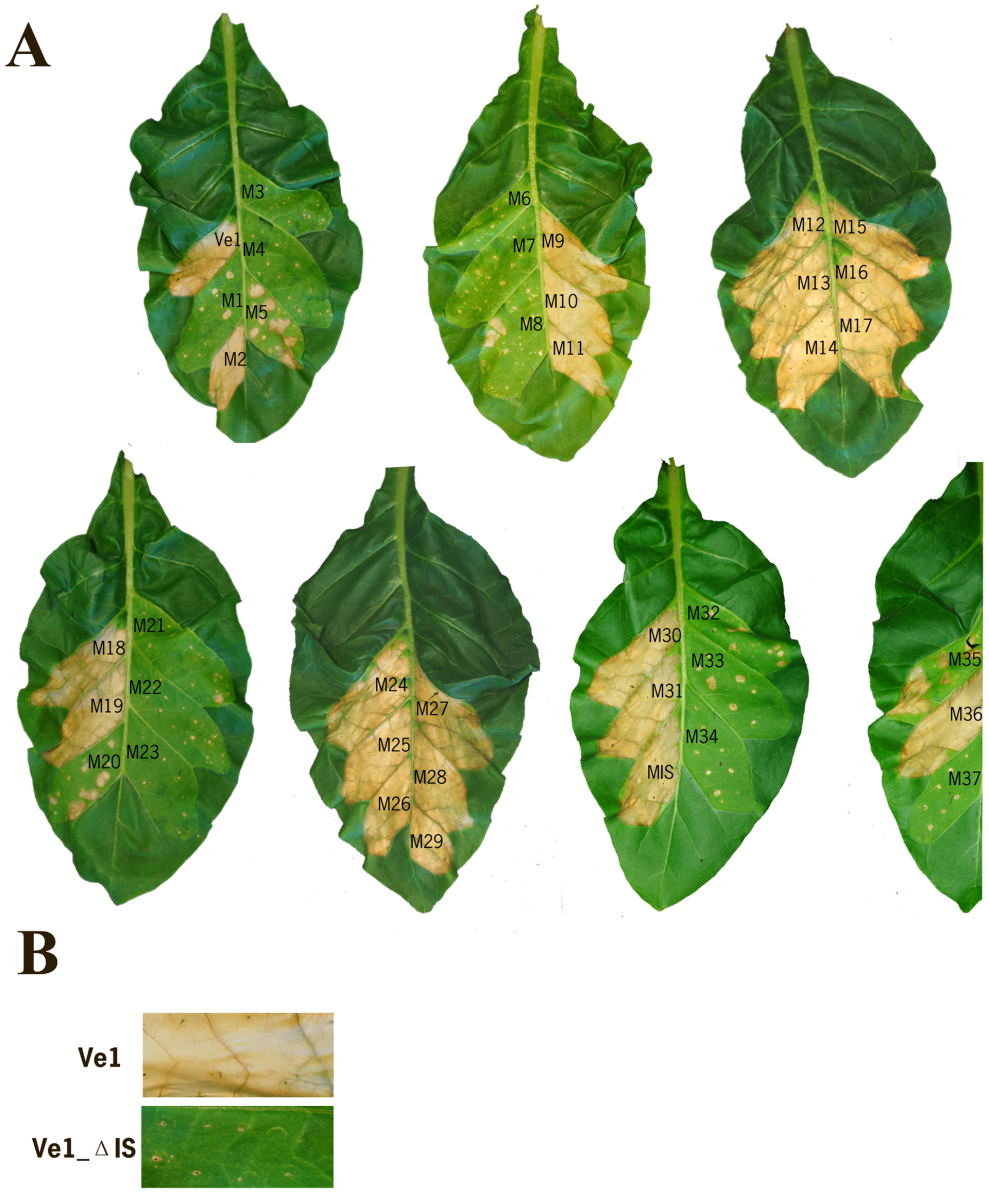
Typical appearance of tobacco leaves transiently co-expressing Ave1 with Ve1 mutant alleles. (**A**) Occurrence of HR upon co-expression of Ave1 and Ve1 double alanine scanning mutant alleles. (**B**) Co-expression of the island domain deletion construct Ve1_ΔIS with Ave1. All pictures were taken at 5 days post infiltration and are representative of at least three independent experiments.

We previously suggested that ligand recognition is determined by the Ve1 eLRRs 1 to 30 [13]. To determine which eLRRs of the C1 domain are required for Ve1 functionality in more detail, tobacco leaves were co-infiltrated with 1∶1 mixture of *Agrobacterium tumefaciens* cultures carrying *Ave1* and *Ve1* alleles that encode mutants in the C1 domain (M1–M31). Intriguingly, agroinfiltration in at least three independent experiments revealed that expression of mutant alleles M1, M3 to M8, and M20 to M23 together with Ave1 showed significantly compromised HR at five days post infiltration (dpi; Figure 2; Figure 3A). In contrast, co-expression of Ave1 with the mutant alleles M2, M9–M19, and M24–M31 resulted in full HR. To exclude the possibility that co promised HR is the result of the expression of unstable receptor proteins rather, the Ve1 mutants that failed to induce full HR were C-terminally tagged with a green fluorescent protein (GFP), and protein stability was verified by immunoblotting (Figure S1). Similar to the discrepancies have previously been reported for Ve1, Ve2 and other eLRR proteins, the estimated sizes of the Ve1-GFP proteins exceeded the calculated sizes, likely due to N-glycosylation of the proteins [13], [22], [23]. Importantly, most of the GFP-tagged Ve1 mutants accumulated to similar levels as GFP-tagged wild type Ve1 protein or GFP-tagged Ve1 mutant M2 that are able to induce full HR. Only mutant M1-GFP could not be detected by western blotting, indicating that this LRR are essential for Ve1 protein stability (Figure S1).

To further assess functionality of the mutant alleles, all mutant constructs were transformed into Arabidopsis [24]. For each mutant, three independent transformants were challenged with race 1 *V. dahliae*. As expected based on the occurrence of HR in tobacco, transgenic plants carrying the non-functional mutant alleles M1, M3–M8 and M20–M23 displayed Verticillium wilt symptoms that were comparable to those on inoculated non-transgenic control plants (Figure 2; Figure S2). In contrast, expression of functional mutant alleles M2, M9–M19 and M24–M31 in Arabidopsis resulted in complete *Verticillium* resistance, as the transgenes showed few to no symptoms upon inoculation when compared to non-transgenic control plants (Figure 2; Figure S2). The differential symptom display correlated with the amount of *Verticillium* biomass, when compared with the *Verticillium* biomass in inoculated wild type plants and Ve1-expressing plants (Figure 2). Collectively, these results show that the LRR region between eLRR1 and eLRR8, as well as between eLRR20 and eLRR23, is required for Ve1-mediated resistance.

### The island (C2) domain is required for Ve1 function

To test the contribution of the island domain, the non-LRR region (C2) that separates the two LRR-containing domains (C1 and C3) in the extracellular domain of Ve1, to Ve1 function, two alanine substitutions were introduced into the predicted island domain to engineer mutant allele MIS (Figure 1). Agroinfiltraion revealed that the mutant allele can still activate an HR upon co-expression with Ave1, as the complete infiltrated sectors became fully necrotic (Figure 2; Figure 3A). Similarly, expression of the mutant allele in Arabidopsis resulted in *Verticillium* resistance, as the transgenes showed few to no symptoms of disease and significantly less fungal biomass accumulated upon inoculation with race 1 *V. dahliae* when compared with wild-type plants (Figure 2; Figure S2). Previously, Wang et al. [25] demonstrated that deletion of the island domain from CLV2 does not affect its functionality in plant development. We thus designed the deletion construct Ve1_ΔIS, in which the complete island domain of Ve1 was removed. In contrast to mutant allele MIS, co-expression of the deletion construct with Ave1 did not induce an HR in tobacco (Figure 3B), suggesting that the island domain is required for Ve1 functionality. Importantly, the Ve1_ΔIS-GFP mutant accumulates to detectable levels (Figure S1).

### Alanine scanning reveals functionally important solvent-exposed residues in the β-strands of the C3 domain

Based on domain swaps between Ve1 and Ve2, we previously demonstrated that the C3 domain and C-terminus of Ve2 are not able to activate immune signaling [13]. To further determine the role of solvent exposed residues in the β-strands of the C3 domain, tobacco leaves were co-infiltrated with *A. tumefaciens* cultures carrying mutant *Ve1* alleles in the region that encodes the C3 domain (M32-M37) and Ave1. Intriguingly, five of the six Ve1 mutants that were generated in the C3 domain resulted in abolished or significantly compromised HR in tobacco leaves at five dpi, as only mutant (M36) still activated full HR (Figure 2; Figure 3A). The nonfunctional mutants were C-terminally tagged with GFP, and protein stability was tested by immunoblotting (Figure S1). GFP-tagged mutant proteins M32-GFP, M35-GFP and M37-GFP were found to accumulate to similar levels as non-mutated Ve1-GFP protein or the functional mutant protein M36-GFP, whereas the M32-GFP and M34-GFP mutant constructs did not lead to detectable protein levels, suggesting that these LRRs are essential for Ve1 protein stability (Figure S1). As expected based on the agroinfiltration results, expression of M36 resulted in *Verticillium* resistance in Arabidopsis, while plants expressing the other C3 domain mutant alleles displayed typical Verticillium wilt symptoms that were comparable to wild type plants (Figure 2; Figure S2). Collectively, as expected based on the domain swaps experiments [13], these alanine scanning assays confirm that the C3 region (eLRR32-eLRR37) is critical for Ve1 functionality.

### The C3 domain of Cf-9 is required for functionality

**Figure 4:**
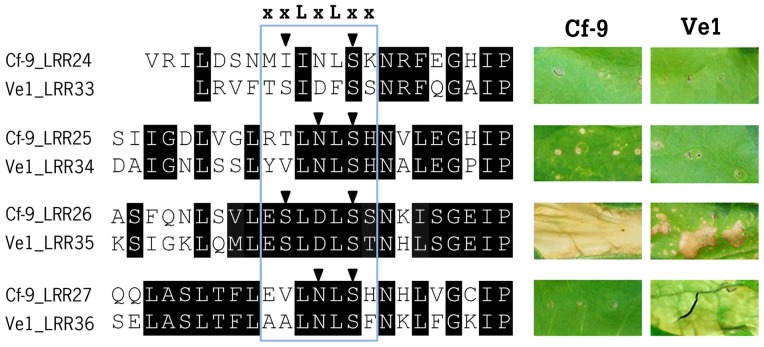
The C3 domain of Cf-9 is required for functionality. A sequence alignment of the C3 domain of Cf-9 and Ve1 is shown, with identical and similar residues indicated with black shading. The putative parallel β-strands (xxLxLxx) on the concave surface are boxed. Triangles represent solvent-exposed amino acid residues subjected to alanine substitution. Functional characterization of the mutants is shown on the right. Photographs illustrate typical appearance of tobacco leaves upon co-expression of Cf-9 mutants with Avr9, or Ve1 mutants with Ave1. Pictures were taken at 5 days post infiltration and are representative of at least three independent experiments.

Previous comparison of eLRR-RLP sequences of Arabidopsis, rice and tomato has shown that the C3 domains of these proteins are relatively conserved. Based on this finding it was suggested that the conserved C3 region may be involved in interaction with common factors, such as (a) co-receptor(s) [11]–[13], [26]. To prove that the C3 domain of Cf-9 is functionally important similar to that of Ve1, we performed site-directed mutagenesis on the C3 domain of Cf-9, which has four eLRRs. The alanine substitutions are made at the same sites of the concave surface that were used for the mutagenesis of Ve1 (Figure 4). Intriguingly, co-expression of Avr9 with Cf-9 mutants M24, M25 and M27 resulted in compromised HR, whereas co-expression with mutant M26 did not show compromised HR. Collectively, these results demonstrate that the C3 region is required for Cf-9 function, as was similarly demonstrated for Ve1.

### Alanine scanning of putative functional motifs in the C-terminus of Ve1

In addition to the eLRR domain, the domain swaps between Ve1 and Ve2 also pointed towards a function of the transmembrane region and cytoplasmic tail of Ve1 [13]. A GxxxG motif that has been implicated in protein-protein interactions is found in the transmembrane domain of many membrane proteins [27], [28], including Ve1 and other eLRR-containing cell surface receptors such as Cf-2, Cf-4, Cf-9, EFR and HrcVf [11]. Interestingly, a mutation in the second glycin of GxxxG motif abolished the function of Cf-9, which was thought to be due to disruption of the interaction with a co-receptor that associates through the GxxxG motif [29]. Similar mutations in Arabidopsis AtRLP51 and AtRLP55 resulted in constitutively activated defense [30]. Furthermore, endocytosis of membrane proteins is often associated with presence of a Yxxφ or E/DxxxLφ consensus motif in the cytoplasmic domains of such proteins, where φ is a hydrophobic residue and x is any amino acid [31], [32]. Both Yxxφ and E/DxxxLφ motifs are present in the cytoplasmic domain of Ve1. To further determine the role of the GxxxG, E/DxxxLφ and Yxxφ motifs in Ve1 function, we employed alanine scanning mutagenesis.

### The putative transmembrane GxxxG motif is not required for Ve functionality

**Figure 5:**
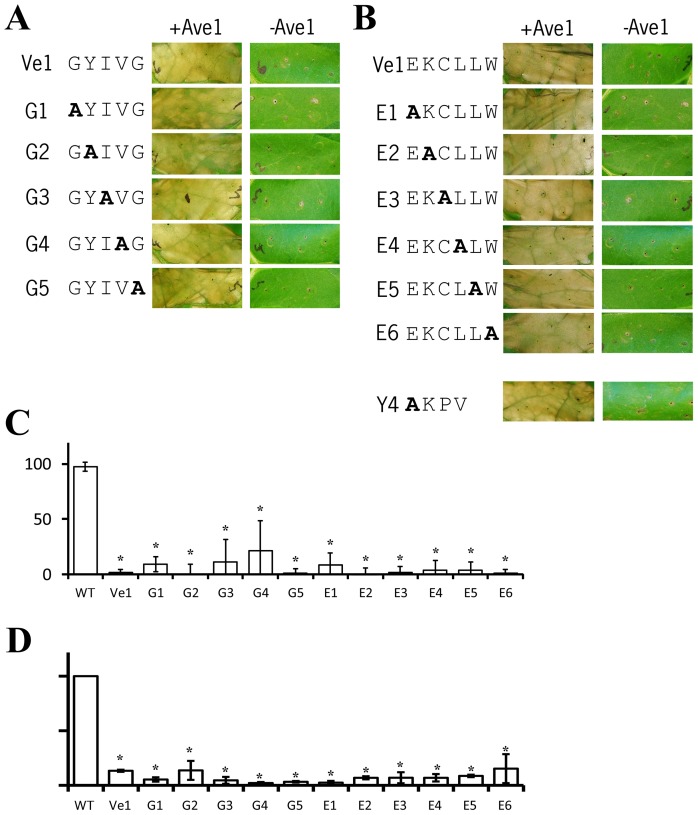
The putative transmembrane GxxxG motif and C-terminal endocytosis motifs are not required for Ve1 functionality. (**A**) Typical appearance of tobacco leaves transiently expressing wild type Ve1 and Ve1 mutants in presence or absence of Ave1 for the GxxxG motif (**A**) or the C-terminal endocytosis motifs (**B**). Pictures were taken at 5 days post infiltration and are representative of at least three independent experiments. (**C**) Quantification of *Verticillium* wilt symptoms in wild type (WT) and transgenic lines. Bars represent quantification of symptoms presented as percentage of diseased rosette leaves with standard deviation. WT is set to 100%. Asterisks indicate significant differences when compared with WT (P<0.001). (**D**) Quantification of *Verticillium* biomass in Arabidopsis expressing Ve1 mutants in the GxxxG motif and the C-terminal endocytosis motifs. Fungal biomass determined by real-time qPCR in wild-type (WT) Arabidopsis and transgenic lines, and the fungal biomass in WT plants is set to 100%. For qPCR, *Verticillium* internal transcribed spacer (ITS) transcript levels are shown relative to Arabidopsis RuBisCo transcript levels (for equilibration). Bars represent an average *Verticillium* quantification of three independent transgenic lines. Error bars represent standard deviations of qPCR results from three independent transgenic lines. Asterisks indicate significant differences when compared with WT (P<0.05).

All five residues in the Ve1 putative GxxxG domain were selected for mutagenesis and subjected to alanine substitution (G1 to G5; Figure 5A). Co-expression of the mutants with Ave1 in tobacco showed that the mutations did not affect Ve1 functionality, as full HR was still observed (Figure 5A). Next, Arabidopsis plants were transformed with the mutant alleles, and the resulting transgenes were challenged with *V. dahliae*. As expected, all mutant *Ve1* alleles still mediated *Verticillium* resistance as the transgenic plants showed few to no symptoms upon inoculation and accumulated significantly less fungal biomass when compared with non-transgenic wild type plants (Figure 5C; Figure S3).

### Putative C-terminal endocytosis motifs are not required for Ve1 functionality

To investigate whether the putative C-terminal E/DXXXLφ endocytosis motif is involved in Ve1 functionality, we generated six Ve1 mutant alleles, E1 to E6, in which each amino acid of the E/DXXXLφ motif was replaced by an alanine (Figure 5B). Expression of none of the mutant alleles resulted in reduced HR upon co-expression with Ave1 by agroinfiltration in tobacco (Figure 5B). Also in this case, Arabidopsis transgenes expressing the mutant alleles were resistant against *Verticillium* (Figure 5; Figure S3). Similarly, we generated alanine substitution construct Y4 in which the conserved Tyr1032 of the putative Yxxφ endocytosis motif was mutated. However, co-infiltration with Ave1 showed that also this mutation does not affect Ve1 functionality (Figure 5B). Collectively, although our data do not show whether or not endocytosis of the Ve1 immune receptor takes place as part of the immune signaling process, we show that the two putative endocytosis motifs in the Ve1 C-terminus are not required for Ve1 functionality.

## Discussion

The plant eLRR-containing cell surface receptors encompass many members that were shown to play important roles in either development or pathogen immunity. Since solved structures of receptor-ligand co-crystals often are not readily available, thus far, knowledge about the functioning of plant eLRR receptors is mainly based on domain swaps, domain deletions, gene shuffling analyses and site-directed mutagenesis. We previously swapped domains of Ve1 with homologous domains of its non-functional homolog Ve2, and analysis of the chimeras suggested that Ve2 may still detect the (activity of the) Ave1 effector in the C1 eLRR domain, but that its C3 domain and C-terminus are not able to activate defense signaling. Here, we employed a site-directed mutagenesis strategy to further dissect functional determinants of Ve1.

Previously, site-directed mutagenesis has been employed for functional analysis of eLRR-containing cell surface receptors. For example, van der Hoorn et al [23] analyzed a number of site-directed mutants of Cf-9 and demonstrated that conserved Trp and Cys residues present in the N- and C-terminal eLRR flanking regions are important for Cf-9 activity. Similarly, recently reported site-directed mutations proved that the Cys residues in the N-terminal flanking region of the FLS2 eLRRs are required for protein stability and function [33]. However, as these Trp or Cys residues are conserved in many other plant eLRR proteins as well, they likely contribute to the conformation and stability of the protein rather than to ligand specificity. In addition, another site-directed mutagenesis strategy focused on putative *N*-linked glycosylation sites, which frequently occur in the eLRR domain of cell surface receptors. Through Asn to Asp substitution, van der Hoorn et al [23] demonstrated that four glycosylation sites contribute to Cf-9 functionality. These four sites are located in putative α-helixes that are exposed at the convex surface of the Cf-9 eLRR domain and are also conserved in many plant eLRR proteins [23]. Glycosylation may contribute to protein conformation, facilitate interactions with the cell wall [34], or protect proteins from degradation [35]. However, it seems unlikely that these putative glycosylation sites contribute to ligand specificity of Cf-9 [23]. Most of the Ve1 glycosylation sites are located at convex face of the eLRR domain (18 of 21 for Cf-9 and 15 of 18 for Ve1), and thus they were not specifically targeted in our study. To the best of our knowledge, no examples of ligand perception at convex side of the eLRR domain have been reported [11]. Moreover, *N*-linked glycosylation was determined to make only subtle quantitative contributions to FLS2 functionality [33]. In contrast, alanine scanning mutagenesis on the concave β-sheet surface across the Arabidopsis FLS2 eLRR domain identified eLRR9-eLRR15 as contributors to flagellin perception [36]. To identify eLRRs that are required for Ve1 ligand recognition, we focused our attention on the concave β-sheet surface and evaded conserved hydrophobic leucine residues in β-sheets that are likely involved in framework of protein. A double-alanine scanning was performed in which two of the five variable, solvent exposed residues in a single eLRR repeat were mutated. Mutagenesis of two non-adjacent amino acids increases the chance of substituting functionally important residues.

In this study, we showed that mutant alleles that reveal compromised Ve1 function are restricted to three consecutive eLRR regions, eLRR1-eLRR8, eLRR20-eLRR23 and eLRR32-eLRR37. This is consistent with previously studies, in which eLRR function was found to be determined by solvent-exposed residues in clustered LRRs of the concave β-sheet surface. For example, domain swaps of tomato Cfs revealed that eLRR13-eLRR16 of Cf-4 contribute to ligand specificity [37], while ligand specificity of Cf-9 is determined by eLRR10-eLRR16 [38]. In addition, photoaffinity labelling showed that BAM1 directly interacts with the small peptide ligand CLE9 at the eLRR6–eLRR8 region [39]. Finally, the crystal structure of PGIP showed that the concave surface of eLRR4-eLRR8 is involved in polygalacturonase binding [17]. Similarly, crystallographic studies revealed that brassinosteroid binds to a hydrophobic groove of BRI1 in between the island domain and the concave β-sheet surface of eLRR20-eLRR25 [18], [19]. Significantly, crystal structure analysis showed that flg22 binds to the concave surface of FLS2 eLRR3 to eLRR16 [21]. This similarly holds true for the eLRR domain of mammalian TLRs, for example, a crystal structure of the TLR4–MD-2–LPS complex demonstrated that the TLR4 interaction with cofactor MD-2 is restricted to the concave β-sheet surface of two eLRR clusters, eLRR2-eLRR5 and eLRR8-eLRR10 [40].

Because ligand specificity is often determined by the C1 domain, we previously suggested that this may similarly be true for Ve1 [13]. Therefore, the two regions eLRR1-eLRR8 and eLRR20-eLRR23 are proposed to contribute to ligand binding. However, most of the mutant alleles in the C3 domain (eLRR32-eLRR37) also abolished Ve1 function. This finding is consistent with previous domain swap experiments between Ve1 and Ve2, which demonstrated that the C3 domain of Ve2 is not able to activate successful immune signaling [13]. Similar to Ve1, alanine scanning of the C3 domain of Cf-9, which is rather conserved when compared with the C3 domain of Ve1, compromised its functionality. This is also consistent with previous mutagenesis studies on Cf-9, where Wulff et al [29] showed that the Ser675Leu mutation in the solvent-exposed resides of the concave side of the Cf-9 eLRR24 in the C3 domain abolished functionality. Similarly, van der Hoorn et al [23] proved that Cf-9 function is compromised upon Asp substitution of Asn697, which is located on the concave side of eLRR25. In addition, a Glu662Val mutation in Cf-4 similarly showed the importance of concave side of the eLRR C3 domain [29]. It has previously been demonstrated that the C3 domains of the Cf-4 and Cf-9 receptors, that perceive sequence-unrelated effector proteins Avr4 and Avr9, respectively, is identical, supporting a role in immune signaling rather than in ligand perception [37].

The eLRR domain has recently been shown to be involved in hetero-dimerization of receptor molecules [41]-[43]. Possibly, the relatively conserved C3 domain [11], [13], [26] is involved in the interaction with downstream signaling partners such as (a) common co-receptor(s) [13]. BRASSINOSTEROID INSENSITIVE 1-ASSOCIATED KINASE 1 (BAK1) is such a common co-receptor and forms a heteromerization with FLS2 for activation of plant immunity. Interestingly, although FLS2 do not carry a non-eLRR island domain that interrupts its 28 eLRRs into the C1 and C3 regions, recent crystallographic analysis on FLS2-BAK1-flg22 co-crystals reveals that flg22 ligand binds to the N-terminus of FLS2 (eLRR3-eLRR16), whereas BAK1 binds to concave surface of the C-terminal eLRRs of FLS2 (eLRR18-eLRR25) [21]. Previously, BAK1 was shown to be genetically involved in Ve1-mediated immunity [9], [24]. Other common co-receptor candidates for both Ve1 and Cf proteins have recently been identified as SOBIR1 and SOMATIC EMBRYOGENESIS RECEPTOR-LIKE KINASE 1 (SERK1), which both encode an eLRR-RLK with a short eLRR domain [20], [43]. It was demonstrated that tomato SOBIR1 physically interacts with various eLRR-RLPs, including Cf-9, Cf-4 and Ve1, irrespective of ligand binding [13], [14], while SERK1 was shown to be genetically required for both Ve1- and Cf-4-mediated immune signaling [9], [24]. Although it remains unknown how various eLRR-RLPs interact with SOBIR1 and SERK1, the relatively high conservation of the C3 domain suggests that this region may be involved.

Overall, this study identified exposed concave β-sheet surfaces with a functional role in Ve1-mediated resistance. This extensive analysis of Ve1 provides fuel for our understanding of eLRR protein function and brings novel leads for further research on eLRR protein function in plants.

## Materials and Methods

### Plant materials

Tobacco (*Nicotiana tabacum* cv. Petite Havana SR1) and Arabidopsis (*Arabidopsis thaliana*) plants were grown in the greenhouse at 21°C/19°C during 16/8 hours day/night periods, respectively, with 70% relative humidity and 100 W•m^−2^ supplemental light when the light intensity dropped below 150 W•m^−2^. After agroinfiltration, plants were grown in the climate room at 22°C/19°C during 16/8 hours day/night periods, respectively, with 70% relative humidity. Arabidopsis transformations were performed as described [44]. Homozygous single insert transgenic lines were selected by analyzing the segregation of antibiotic resistance.

### Generation of constructs for over-expression of Ve1 and Cf-9

The tomato *Ve1* coding sequence was PCR amplified from *pMOG800::Ve1* [9] using primers attB-Ve1-F and attB-Ve1-R containing AttB1 and AttB2 sites for Gateway-compatible cloning. The tomato Cf-9 coding sequence was PCR amplified from *pMOG800::Cf-9* [45] using primers attB-Cf9-F and attB-Cf9-R. The resulting PCR product was cleaned from 1% agarose gel using the QIAquick Gel Extraction Kit (Qiagen, Valencia, California) and transferred into donor vector *pDONR207* using Gateway BP Clonase II enzyme mix (Invitrogen, Carlsbad, California) to generate entry vector *pDONR207::Ve1* and *pDONR207::Cf-9*, respectively. The entry constructs *pDONR207::Ve1* and *pDONR207::Cf-9* were subsequently cloned into Gateway destination vector using Gateway LR Clonase II enzyme mix (Invitrogen, Carlsbad, California) to generate expression constructs driven by the CaMV35S promoter. The expression constructs were transformed into *E. coli* and transformants were checked by colony PCR analysis using primers AttB1F and AttB2R. The expression constructs were subsequently sequenced and transformed into *Agrobacterium tumefaciens* strain GV3101 by electroporation.

### Alanine scanning mutagenesis

For the alanine scanning mutagenesis, inverse PCR was performed to introduce alanine substitutions. Primers to introduce mutations (Table S1) were designed according to user manual of GeneTailor site-directed mutagenesis kit (Invitrogen, Carlsbad, California). PCR reactions were performed in a total volume of 30 µL with 23 µL water, 3 µL 10x PCR buffer, 1 µL dNTPs, 1 µL of each primer, 1 µL Pfu DNA polymerase (Promega, Madison, Wisconsin) and 1 µL of *pDONR207::Ve1* or *pDONR207::Cf-9*. The PCR consisted of an initial denaturation step of 5 minutes at 95°C, followed by denaturation for 30 sec at 95°C, annealing for 30 sec at 45°C to 55°C, and extension for 14 min at 72°C for 20 cycles, and then a final extension for 20 min at 72°C.The product was purified by QIAquick PCR Purification Kit (Qiagen, Valencia, California), treated with *Dpn*I endonuclease kinase (New England Biolabs, Ipswich, UK), and transformed into DH5α chemically competent cells. Mutant plasmid DNA was extracted and sequenced to verify the mutations, and recombined with the Gateway-compatible destination vector to generate an expression construct driven by the constitutive CaMV35S promoter.

### *Agrobacterium tumefaciens*-mediated transient expression

*A. tumefaciens* containing expression constructs were infiltrated into tobacco plants as described previously [45]–[47]. Briefly, an overnight culture of *A. tumefaciens* cells was harvested at OD_600_ of 0.8 to 1 by centrifugation and resuspended to a final OD of 2. *A. tumefaciens* cultures containing constructs to express *Ave1* and mutated *Ve1* proteins were mixed in a 1∶1 ratio and infiltrated into leaves of five- to six-week-old tobacco plants. At five days post infiltration (dpi), leaves were examined for necrosis.

### Protein extraction and immunoblotting

For detection of Ve1 mutants that showed compromised function, corresponding mutant constructs were C-terminally tagged with the green fluorescent protein (GFP) as described previously [46]. *A. tumefaciens* containing the relevant expression constructs was infiltrated into tobacco plants as described previously [46]. Tobacco leaves were harvested at two days post infiltration, flash frozen and ground to a fine powder in liquid nitrogen. Total proteins were dissolved in extraction buffer (150 mM Tris-HCL pH 7.5, 150 mM NaCl, 10 mM DTT, 10% glycerol, 10 mM EDTA, 1% IGEPAL CA-630, 0.5% polyvinylpyrrolidon and 1% protease inhibitor cocktail [Roche, Basel, CH]). The immunopurifications and immunoblotting were performed as described previously [48].

### *Verticillium* inoculations

Race 1 *V. dahliae* strain JR2 was grown on potato dextrose agar (PDA) at 22°C. *V. dahliae* conidia were harvested from 7- to 14-day-old fungal plates and washed with tap water. The conidia were suspended to a final concentration of 10^6^ conidia per milliliter in potato dextrose broth (PDB). For inoculation, 2- to 3-week-old Arabidopsis plants were uprooted, and subsequently the roots were dipped in the conidial suspension for 3 min. As a control, plants were mock-inoculated in PDB without conidia. After inoculation, plants were immediately transplanted to new pots, and disease development was evaluated at 21 days post inoculation (dpi) as described earlier [24]. Fungal biomass quantification in infected Arabidopsis plants was performed with real-time quantitative PCR (qPCR) as described previously [49]. Briefly, qPCR was conducted on total DNA isolated from *V. dahliae* infected Arabidopsis with primers amplifying *Verticillium* internal transcribed spacer (ITS; ITS1-F and STVe1-R) and the primers amplifying the Arabidopsis RuBisCo gene as endogenous control (AtRub-F3 and AtRub-R3). The qPCR was conducted using an ABI7300 PCR machine (Applied Biosystems, Foster City, California) in combination with the SensiMix SYBR Hi-ROX Kit (Bioline, London, UK). Real-time PCR conditions were as follows: an initial 95°C hot start activation step for 10 min was followed by denaturation for 15 sec at 95°C, annealing and extension for 60 sec at 60°C for 40 cycles.

## Supporting Information

Figure S1 Stability of Ve1 mutants that showed compromised HR-inducing capacity. GFP-tagged Ve1 mutants were detected by immunoblotting using GFP antibody (α-GFP). Coomassie-stained blots (CBS) showing the 50 kDa Rubisco band present in the input samples confirm equal loading.
(DOCX)

Figure S2 Typical appearance of non-transgenic Arabidopsis (WT) and transgenic Arabidopsis expressing Ve1 mutants, upon mock-inoculation or inoculation with race 1 *V. dahliae*. Pictures were taken at 21 days post inoculation and are representative of three independent experiments.
(DOCX)

Figure S3 Typical appearance of non-transgenic Arabidopsis (WT) and transgenic Arabidopsis producing Ve1 mutants in the putative GxxxG motif and the E/DxxxLφ endocytosis motifs, upon mock-inoculation or inoculation with *V. dahliae* race 1. Pictures were taken at 21 days post infiltration and are representative of three independent experiments.
(DOCX)

Table S1 Primers used in this study.
(DOCX)
