# Correction: Mutational Analysis of the Ve1 Immune Receptor That Mediates *Verticillium* Resistance in Tomato

**DOI:** 10.1371/journal.pone.0220402

**Published:** 2019-07-23

**Authors:** Zhao Zhang, Yin Song, Chun-Ming Liu, Bart P. H. J. Thomma

Following publication of this article [1], the following concerns were noted in Fig 5:

- Fig 5A G1 -Ave1 panel and Fig 5B E1 -Ave1 panel contain a region of overlap;
- Fig 5A G2 -Ave1 panel and Fig 5B E2 -Ave1 panel contain a region of overlap;
- Fig 5A G3 -Ave1 panel and Fig 5B E3 -Ave1 panel contain a region of overlap;
- Fig 5A G4 -Ave1 panel and Fig 5B E4 -Ave1 panel contain a region of overlap;
- Fig 5A G5 -Ave1 panel and Fig 5B E5 -Ave1 panel contain a region of overlap.

**Fig 5:**
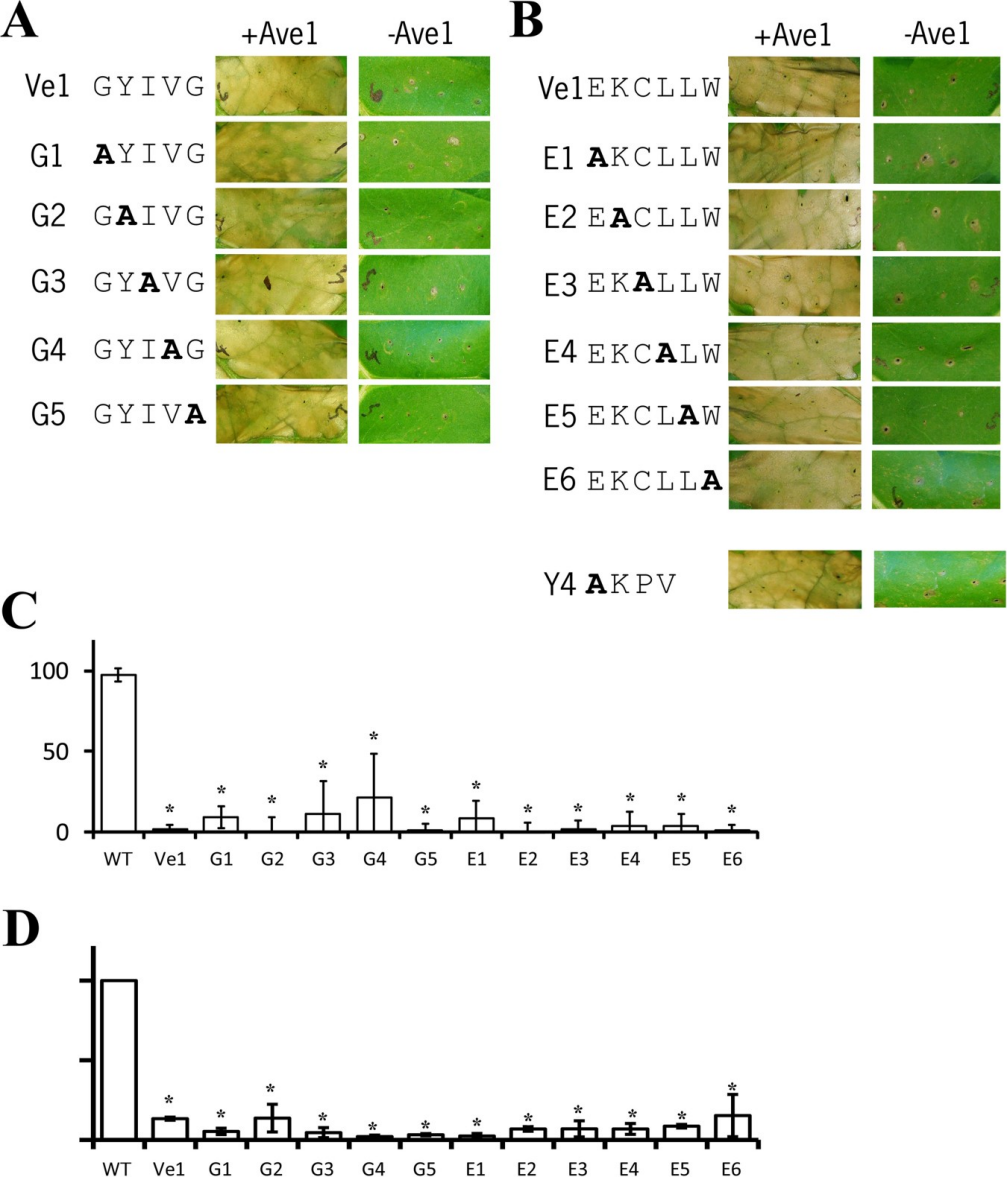
The putative transmembrane GxxxG motif and C-terminal endocytosis motifs are not required for Ve1 functionality. **(A)** Typical appearance of tobacco leaves transiently expressing wild type Ve1 and Ve1 mutants in presence or absence of Ave1 for the GxxxG motif (A) or the C-terminal endocytosis motifs **(B)**. Pictures were taken at 5 days post infiltration and are representative of at least three independent experiments. **(C)** Quantification of *Verticillium* wilt symptoms in wild type (WT) and transgenic lines. Bars represent quantification of symptoms presented as percentage of diseased rosette leaves with standard deviation. WT is set to 100%. Asterisks indicate significant differences when compared with WT (P<0.001). **(D)** Quantification of *Verticillium* biomass in Arabidopsis expressing Ve1 mutants in the GxxxG motif and the C-terminal endocytosis motifs. Fungal biomass determined by real-time qPCR in wild-type (WT) Arabidopsis and transgenic lines, and the fungal biomass in WT plants is set to 100%. For qPCR, *Verticillium* internal transcribed spacer (ITS) transcript levels are shown relative to Arabidopsis RuBisCo transcript levels (for equilibration). Bars represent an average *Verticillium* quantification of three independent transgenic lines. Error bars represent standard deviations of qPCR results from three independent transgenic lines. Asterisks indicate significant differences when compared with WT (P<0.05).

The authors explain that an error during figure preparation led to duplication of figures and apologise for this oversight. The authors provide the uncropped and unadjusted images underlying all panels in Fig 5A and 5B as Supporting Information S1 File. In addition, the authors provide an updated Fig 5 with the correct images with this correction.

The data underlying other figure panels in the article are available upon request from the corresponding author.

A member of the Editorial Board reviewed the updated Fig 5 and confirmed that the changes do not affect the conclusions in the article.

## Supporting information

S1 File Uncropped and unadjusted images underlying all panels of Fig 5A and 5B. (ZIP)
