# Supplementary material for: Mutational Analysis of the Ve1 Immune Receptor That Mediates Verticillium Resistance in Tomato
Source: PLoS One. 2014 Jun 9;9(6):e99511. doi: 10.1371/journal.pone.0099511 (PMC4049777; doi:10.1371/journal.pone.0099511)
Supplement: Figure S1 — Stability of Ve1 mutants that showed compromised HR-inducing capacity. GFP-tagged Ve1 mutants were detected by immunoblotting using GFP antibody (α-GFP). Coomassie-stained blots (CBS) showing the 50 kDa Rubisco band present in the input samples confirm equal loading. (DOCX) [file pone.0099511.s001.docx]

**
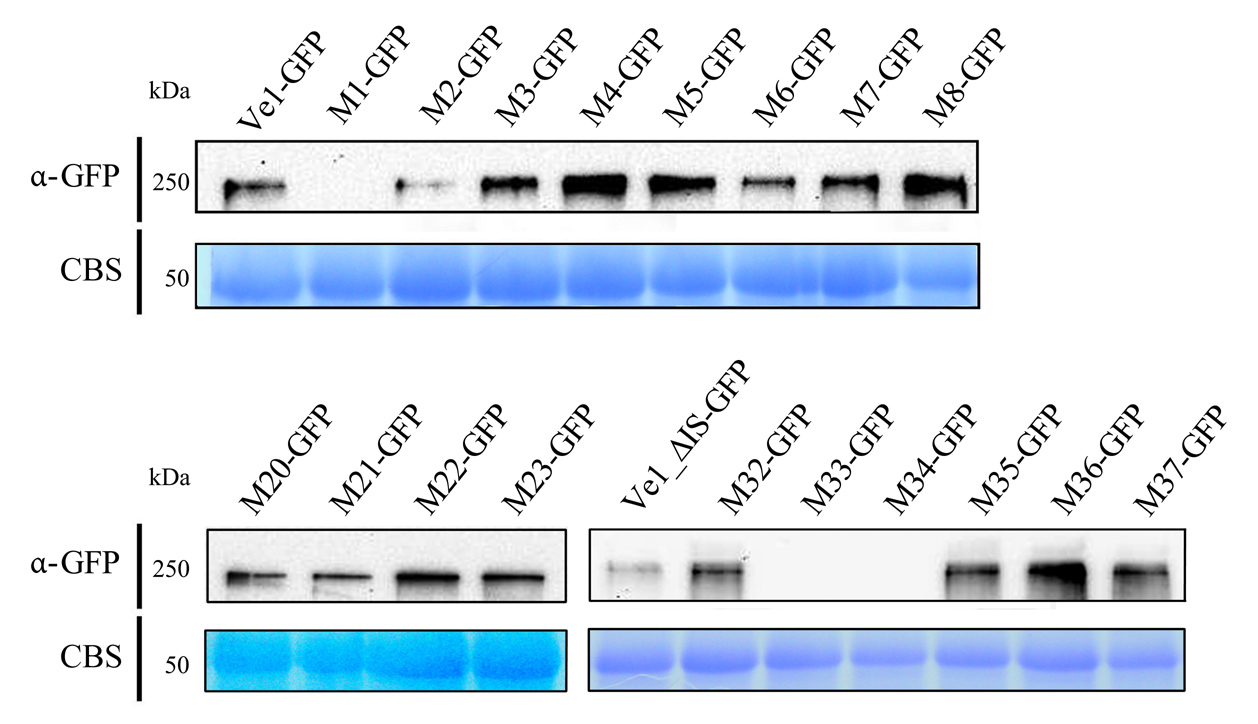
**

**Figure S1.** Stability of Ve1 mutants that showed compromised HR-inducing capacity. GFP-tagged Ve1 mutants were detected by immunoblotting using GFP antibody (α-GFP). Coomassie-stained blots (CBS) showing the 50 kDa Rubisco band present in the input samples confirm equal loading.
