# Supplementary material for: Mutational Analysis of the Ve1 Immune Receptor That Mediates Verticillium Resistance in Tomato
Source: PLoS One. 2014 Jun 9;9(6):e99511. doi: 10.1371/journal.pone.0099511 (PMC4049777; doi:10.1371/journal.pone.0099511)
Supplement: Figure S2 — Typical appearance of non-transgenic Arabidopsis (WT) and transgenic Arabidopsis expressing Ve1 mutants, upon mock-inoculation or inoculation with race 1 V. dahliae. Pictures were taken at 21 days post inoculation and are representative of three independent experiments. (DOCX) [file pone.0099511.s002.docx]

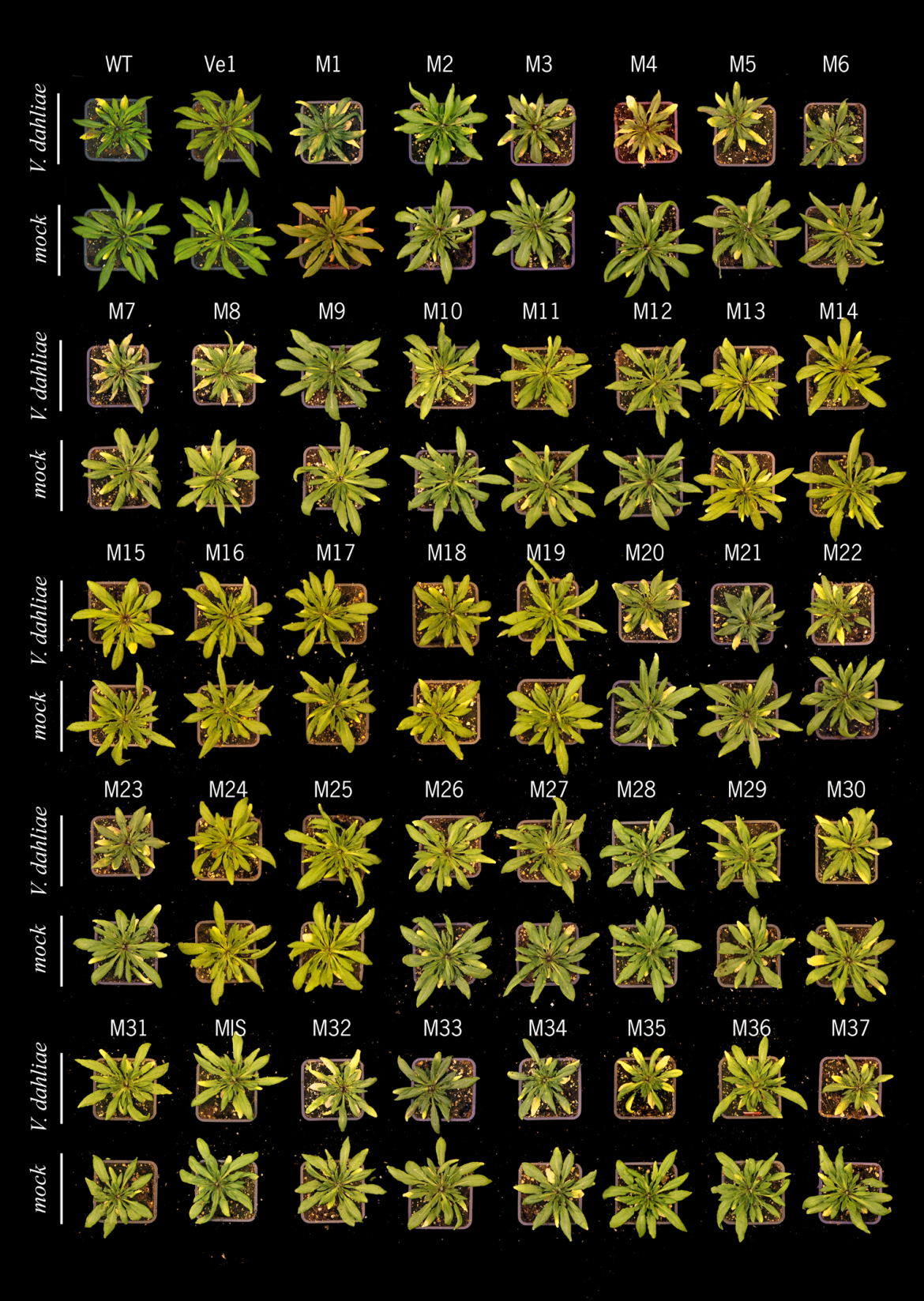


**Figure S2.** Typical appearance of non-transgenic Arabidopsis (WT) and transgenic Arabidopsis expressing Ve1 mutants, upon mock-inoculation or inoculation with race 1 *V. dahliae*. Pictures were taken at 21 days post inoculation and are representative of three independent experiments.
