# Supplementary material for: Mutational Analysis of the Ve1 Immune Receptor That Mediates Verticillium Resistance in Tomato
Source: PLoS One. 2014 Jun 9;9(6):e99511. doi: 10.1371/journal.pone.0099511 (PMC4049777; doi:10.1371/journal.pone.0099511)
Supplement: Figure S3 — Typical appearance of non-transgenic Arabidopsis (WT) and transgenic Arabidopsis producing Ve1 mutants in the putative GxxxG motif and the E/DxxxLφ endocytosis motifs, upon mock-inoculation or inoculation with V. dahliae race 1. Pictures were taken at 21 days post infiltration and are representative of three independent experiments. (DOCX) [file pone.0099511.s003.docx]

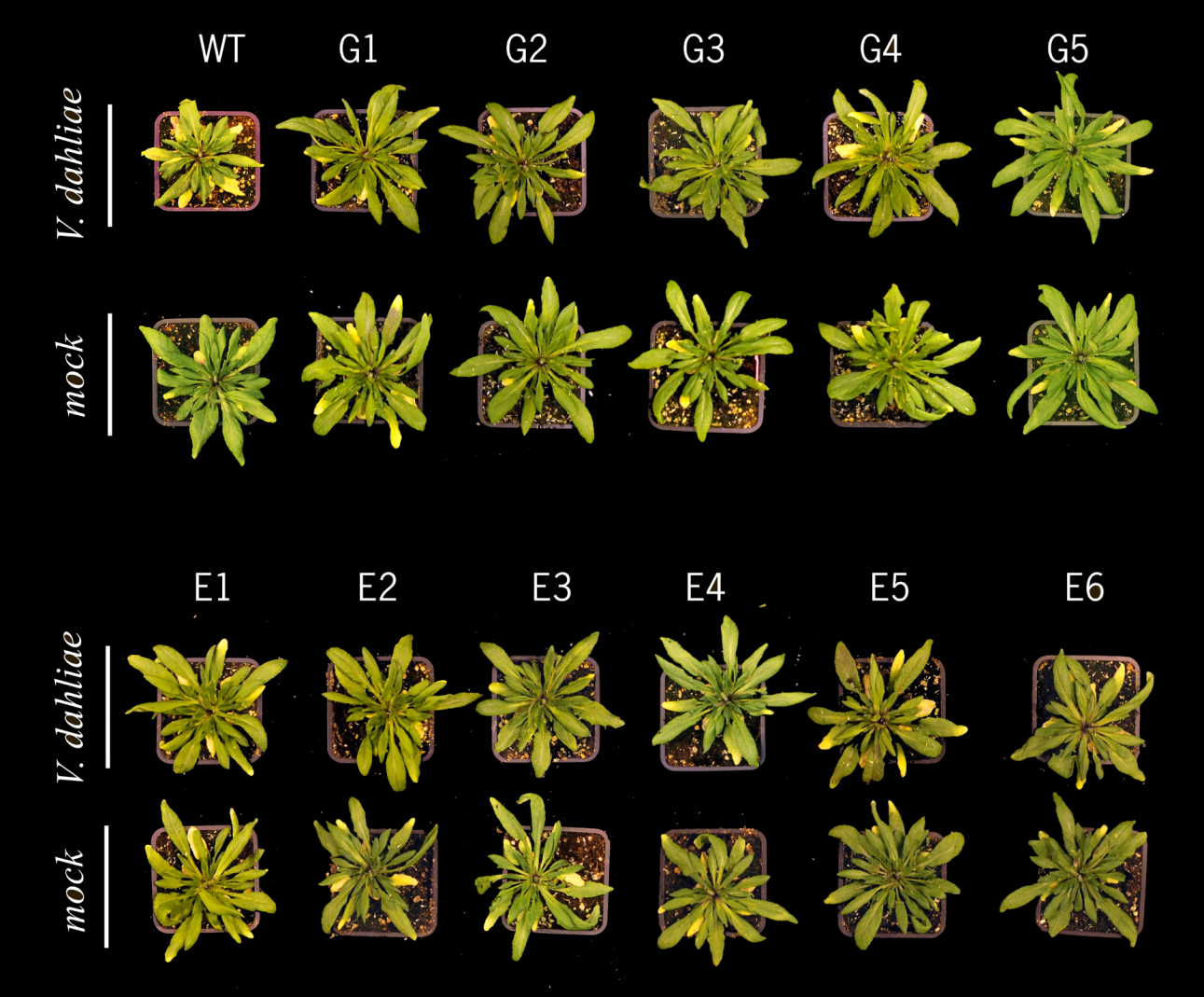


**Figure S3.** Typical appearance of non-transgenic Arabidopsis (WT) and transgenic Arabidopsis producing Ve1 mutants in the putative GxxxG motif and the E/DxxxLφ endocytosis motifs, upon mock-inoculation or inoculation with *V. dahliae* race 1. Pictures were taken at 21 days post infiltration and are representative of three independent experiments.
