# Supplementary material for: Mutational Analysis of the Ve1 Immune Receptor That Mediates Verticillium Resistance in Tomato
Source: PLoS One. 2014 Jun 9;9(6):e99511. doi: 10.1371/journal.pone.0099511 (PMC4049777; doi:10.1371/journal.pone.0099511)
Supplement: Table S1 — Primers used in this study. (DOCX) [file pone.0099511.s004.docx]

**Table S1.** Primers used in this study

| **Primer name** | **Sequence (5’-3’)** |
| --- | --- |
| attB1F | ACAAGTTTGTACAAAAAAGCAGGCT |
| attB2R | ACCACTTTGTACAAGAAAGCTGGGT |
| attB-Ve1-F | GGGGACAAGTTTGTACAAAAAAGCAGGCTATGAAAATGATGGCAACTCT |
| attB-Ve1-R | GGGGACCACTTTGTACAAGAAAGCTGGGTTCACTTTCTTGAAAACCA |
| attB-Cf9-F | GGGGACAAGTTTGTACAAAAAAGCAGGCTATGGATTGTGTAAAACTT |
| attB-Cf9-R | GGGGACCACTTTGTACAAGAAAGCTGGGTCTAATATCTTTTCTTGTG |
| M1F | CCAATATGATGCTACTTTGGCAAAG |
| M1R | TTCTTTGCCAAAGTAGCATCATATTGGAGGC |
| M2F | TCGCTTTGGCACTGGATGCTGAGAC |
| M2R | GTCTCAGCATCCAGTGCCAAAGCGA |
| M3F | TATCTTGAGGCCCTAGCTTTGGCTGACAACATGT |
| M3R | GTCAGCCAAAGCTAGGGCCTCAAGATATTG |
| M4F | GTACCTGGCTTTATCCGCTGCTGGTTTTGTCG |
| M4R | CCAGCAGCGGATAAAGCCAGGTACTTCAAGT |
| M5F | CTAGTTGCTCTTGATCTCTCAGCTATTCTCCCTTT |
| M5R | GAATAGCTGAGAGATCAAGAGCAACTAGCCTTGTT |
| M6F | CTTAGAGCGCTTTACCTTGCTGGGGTTGATCTTTC |
| M6R | ACCCCAGCAAGGTAAAGCGCTCTAAGCTCTGTTGA |
| M7F | AACTTGGCCGTTTTGGCCTTGCGTGATTGTCAAAT |
| M7R | CACGCAAGGCCAAAACGGCCAAGTTAGGCAAATGT |
| M8F | CTTTTGTCGCACTTGACGCGAACAATCTCT |
| M8R | GATTGTTCGCGTCAAGTGCGACAAAAGAGA |
| M9F | CGAACTTGGCTACATTGGCCCTGGGCTCTT |
| M9R | CCCAGGGCCAATGTAGCCAAGTTCGAGAAA |
| M10F | TAGAGGCTTTGGACTTGGCAATTAACAAGTTGC |
| M10R | GTTAATTGCCAAGTCCAAAGCCTCTAAAAC |
| M11F | GGATAGCACTAGCCTACACCAACTTTTCCG |
| M11R | GGTGTAGGCTAGTGCTATCCTCCTCAGAGA |
| M12F | ATCTAGCCAGGTTAGAGCTTGCTAATTGCAATT |
| M12R | GCAATTAGCAAGCTCTAACCTGGCTAGATTTTGA |
| M13F | ATCTTGCTTATTTGGATTTCGCCTTCAACAATTTC |
| M13R | TTGAAGGCGAAATCCAAATAAGCAAGATTTCTAAG |
| M14F | AACTCGCCTACTTAGACCTTGCACGTAATGGTCTA |
| M14R | TTACGTGCAAGGTCTAAGTAGGCGAGTTTCTTGGA |
| M15F | CCACATTGCTTTAGCGAACAATTTACTCAGCGG |
| M15R | ATTGTTCGCTAAAGCAATGTGGACAAGCTC |
| M16F | TTGCAGGCGCTTTTTCTTGCCAGAAATCAA |
| M16R | ATTTCTGGCAAGAAAAAGCGCCTGCAACGA |
| M17F | TGGATGCAGTTGACTTGGCAAACAACCACC |
| M17R | GTTGTTTGCCAAGTCAACTGCATCCAACGG |
| M18F | GTGCTCGCACTTGCTTCCAACTTCTTTAGA |
| M18R | GTTGGAAGCAAGTGCGAGCACCTTAAGCCT |
| M19F | AACCTTGCAAGACTGGAGCTTGCTTACAAT |
| M19R | ATTGTAAGCAAGCTCCAGTCTTGCAAGGTTGCTCA |
| M20F | CATATTGGCATTAGCGTCTGCTCGGCTGCA |
| M20R | CAGCCGAGCAGACGCTAATGCCAATATGTT |
| M21F | ACTTAGCCCTTTCAGCCAACCAAATATTGG |
| M21R | TTGGTTGGCTGAAAGGGCTAAGTGCATCAT |
| M22F | ACCTGGCTCTTGCATTCAATCAGCTGGAGT |
| M22R | TTGAATGCAAGAGCCAGGTGGGTGAGACCT |
| M23F | GTCCTTGCTTTGGCTTCCAACCGTTTAAAA |
| M23R | GTTGGAAGCCAAAGCAAGGACTACAAGATT |
| M24F | GAACTACGCTAGCAATAATTTAGCCAATTCCATCC |
| M24R | GGAATTGGCTAAATTATTGCTAGCGTAGTTCACAT |
| M25F | TTGCCGCCTTTTTCGCGGTAGCAAACAATG |
| M25R | GCTACCGCGAAAAAGGCGGCAAAACCAAGA |
| M26F | TACCTTGCAGTTCTTGATTTCGCTAACAATGCC |
| M26R | CATTGTTAGCGAAATCAAGAACTGCAAGGTAGC |
| M27F | AAACTTGCAGTGCTGAATCTTGCGAACAATAAACT |
| M27R | TTATTGTTCGCAAGATTCAGCACTGCAAGTTTTGT |
| M28F | GCTCTAGCAACATTAGACCTCGCTGCGAATAACTT |
| M28R | ATTCGCAGCGAGGTCTAATGTTGCTAGAGCACAAC |
| M29F | GGTCCTGGCTGTTGGAGCTAACAGACTTGT |
| M29R | GTCTGTTAGCTCCAACAGCCAGGACCTC |
| M30F | CAGTCTGGCGGTCCTAGTCTTGGCCTCCAATAAATTC |
| M30R | ATTGGAGGCCAAGACTAGGACCGCCAGACTG |
| M31F | GATCATAGCTATAGCTGCCAACAACTTCAC |
| M31R | GTTGTTGGCAGCTATAGCTATGATCTGGAG |
| MISF | ATGATGGCTGCAGATGATTACGCGGAGACAGGACGC |
| MISR | GTCTCCGCGTAATCATCTGCAGCCATCATTCCTCTC |
| M32F | CAGTGGCATTAACCATCAAAGCCATGGAGCTGGAGCT |
| M32R | CTCCATGGCTTTGATGGTTAATGCCACTGTGTCCTG |
| M33F | TTCACAGCTATTGATTTCGCTTCCAATAGATTT |
| M33R | ATTGGAAGCGAAATCAATAGCTGTGAAGACCCT |
| M34F | GTTCTGGCTCTGGCACACAATGCCCTTGAGGGACCA |
| M34R | CATTGTGTGCCAGAGCCAGAACATAAAGTG |
| M35F | CTTGAAGCACTAGACCTGGCAACAAACCAC |
| M35R | GTTTGTTGCCAGGTCTAGTGCTTCAAGCAT |
| M36F | GCTTTGGCCTTAGCGTTCAACAAATTGTTT |
| M36R | GTTGAACGCTAAGGCCAAAGCTGCTAAGAA |
| M37F | CTATGCGCGCTCCCTCTCGCCAACAGTTGTCAAAGC |
| M37R | ACTGTTGGCGAGAGGGAGCGCGCATAGGCCACTGTT |
| Cf-9 M24F | CATGGCTATCAATCTCGCTAAGAACAGAT |
| Cf-9 M24R | TTCTTAGCGAGATTGATAGCCATGTTAGA |
| Cf-9 M25F | GTTGGCTTTGGCTCACAATGTC |
| Cf-9 M25R | TGTGAGCCAAAGCCAACGTACG |
| Cf-9 M26F | CGAAGCTTTGGATCTCGCATCTAATAAA |
| Cf-9 M26R | TAGATGCGAGATCCAAAGCTTCGAGTACT |
| Cf-9 M27F | CTTAGCTCTCGCTCACAATCAT |
| Cf-9 M27R | TGTGAGCGAGAGCTAAGACTTCA |
| Cf-9 M24F | CATGGCTATCAATCTCGCTAAGAACAGAT |
| Cf-9 M24R | TTCTTAGCGAGATTGATAGCCATGTTAGA |
| Cf-9 M25F | GTTGGCTTTGGCTCACAATGTC |
| Cf-9 M25R | TGTGAGCCAAAGCCAACGTACG |
| Cf-9 M26F | CGAAGCTTTGGATCTCGCATCTAATAAA |
| Cf-9 M26R | TAGATGCGAGATCCAAAGCTTCGAGTACT |
| Cf-9 M27F | CTTAGCTCTCGCTCACAATCAT |
| Cf-9 M27R | TGTGAGCGAGAGCTAAGACTTCA |
| AtRub-F3 | GCAAGTGTTGGGTTCAAAGCTGGTG |
| AtRub-R3 | CCAGGTTGAGGAGTTACTCGGAATGCTG |
| ITS1-F | AAAGTTTTAATGGTTCGCTAAGA |
| STVe1-R | CTTGGTCATTTAGAGGAAGTAA |
