# Supplementary material for: Correction: Mutational Analysis of the Ve1 Immune Receptor That Mediates Verticillium Resistance in Tomato
Source: PLoS One. 2019 Jul 23;14(7):e0220402. doi: 10.1371/journal.pone.0220402 (PMC6650063; doi:10.1371/journal.pone.0220402)
Supplement: S1 File — (ZIP) [file pone.0220402.s001.zip › Key to Uncropped Images.docx]

For GXXXG:

1. G1+Ave1
2. G2+Ave1
3. G3+Ave1
4. G4+Ave1
5. G5+Ave1
6. Ve1+Ave1
7. Cf-9+Avr9

For GXXXG control (no Ave1):

1. G1
2. G2
3. G3
4. G4
5. G5
6. Ve1
7. Ave1
8. Cf-9

For ExxxLx:

1. Ve1+Ave1
2. E1+Ave1
3. E2+Ave1
4. E3+Ave1
5. E4+Ave1
6. E5+Ave1
7. E6+Ave1

For ExxxLx control (no Ave1):

1. E1
2. E2
3. E3
4. E4
5. E5
6. E6
7. Ve1
8. Ave1
