# Supplementary figures and images for: Correction: Mutational Analysis of the Ve1 Immune Receptor That Mediates Verticillium Resistance in Tomato
Source: PLoS One. 2019 Jul 23;14(7):e0220402. doi: 10.1371/journal.pone.0220402 (PMC6650063; doi:10.1371/journal.pone.0220402)

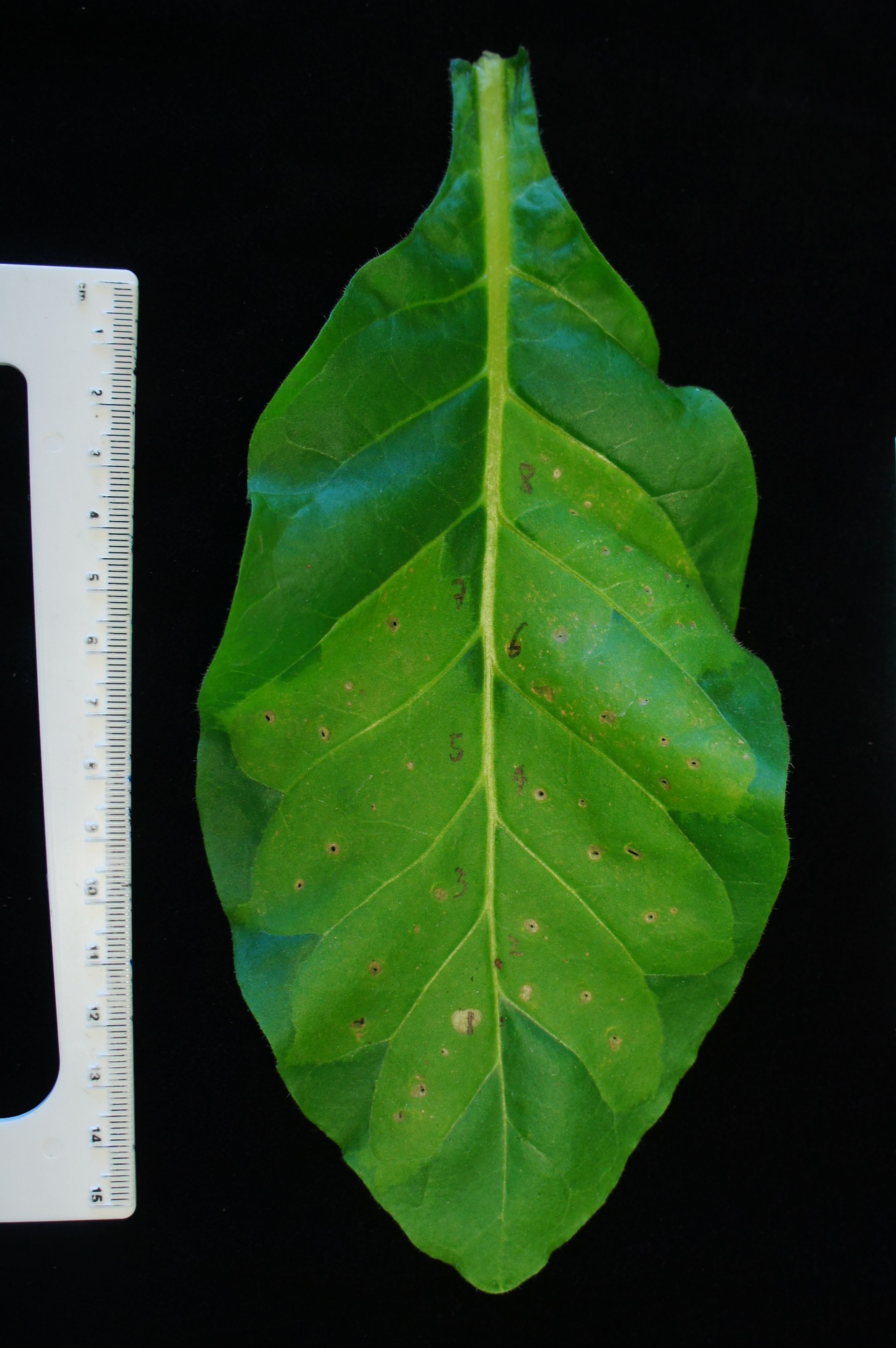

Supplement: S1 File — (ZIP) [file pone.0220402.s001.zip › ExxxLx control.jpg]

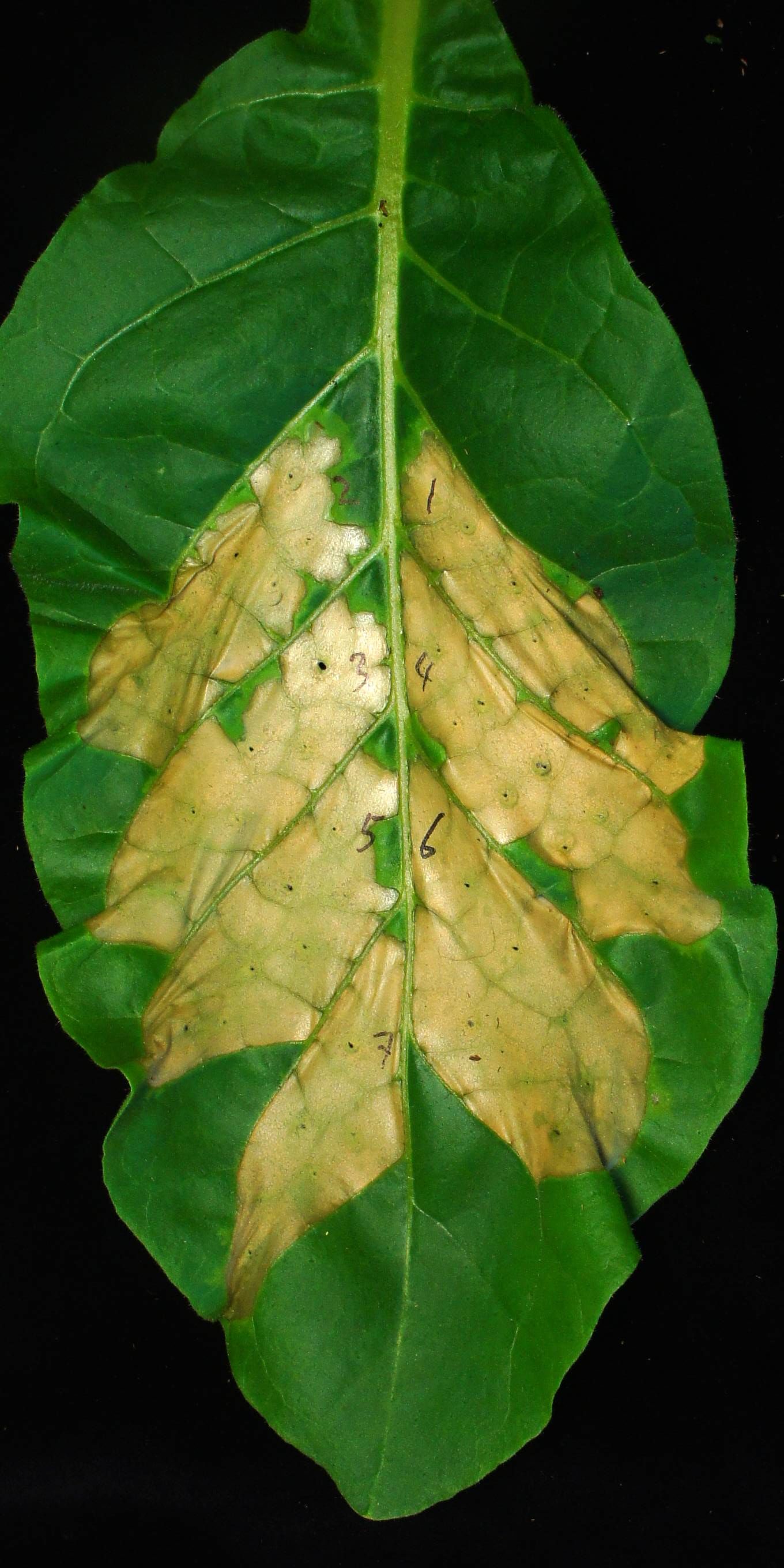

Supplement: S1 File — (ZIP) [file pone.0220402.s001.zip › ExxxLx.jpg]

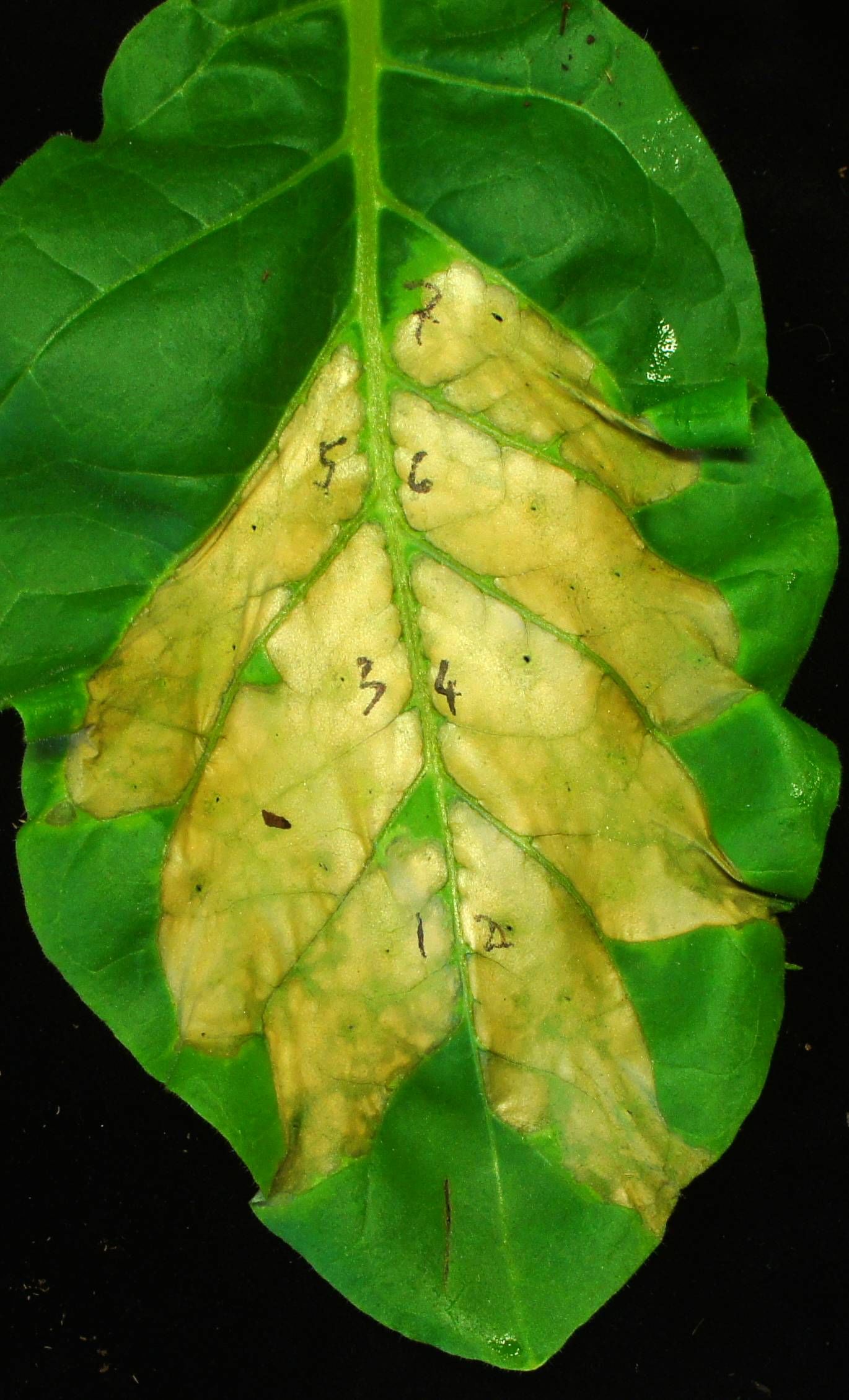

Supplement: S1 File — (ZIP) [file pone.0220402.s001.zip › GXXXG.JPG]
